# Supplementary material for: Unveiling the Bioactive Potential of the Invasive Jellyfish Phyllorhiza punctata Through Integrative Transcriptomic and Proteomic Analyses
Source: Biomolecules. 2025 Aug 4;15(8):1121. doi: 10.3390/biom15081121 (PMC12383608; doi:10.3390/biom15081121)
Supplement: Supplementary file 1 [file biomolecules-15-01121-s001.zip › Suplementary Material/FileS3/PEPFOLD4/XP_065060546_8472/clusters.html]

 PEP-FOLD clusters


Download archive of all models  here   

|  |  |  |
| --- | --- | --- |
| # | file | sOPEP |
| # cluster 1 - Size: 99 |  |  |
|  | XP\_065060546\_8472-model1 | -12.5697 |
|  | XP\_065060546\_8472-model1.1 | -12.5697 |
|  | XP\_065060546\_8472-model1.2 | -12.5697 |
|  | XP\_065060546\_8472-model1.3 | -12.5697 |
|  | XP\_065060546\_8472-model1.4 | -12.5697 |
|  | XP\_065060546\_8472-model1.5 | -12.5697 |
|  | XP\_065060546\_8472-model1.6 | -12.5697 |
|  | XP\_065060546\_8472-model1.7 | -12.5697 |
|  | XP\_065060546\_8472-model1.8 | -12.5697 |
|  | XP\_065060546\_8472-model1.9 | -12.5697 |
|  | XP\_065060546\_8472-model1.10 | -12.5697 |
|  | XP\_065060546\_8472-model1.11 | -12.5697 |
|  | XP\_065060546\_8472-model1.12 | -12.5697 |
|  | XP\_065060546\_8472-model1.13 | -12.5697 |
|  | XP\_065060546\_8472-model1.14 | -12.5697 |
|  | XP\_065060546\_8472-model1.15 | -12.5697 |
|  | XP\_065060546\_8472-model1.16 | -12.5697 |
|  | XP\_065060546\_8472-model1.17 | -12.5697 |
|  | XP\_065060546\_8472-model1.18 | -12.5697 |
|  | XP\_065060546\_8472-model1.19 | -12.5697 |
|  | XP\_065060546\_8472-model1.20 | -12.5697 |
|  | XP\_065060546\_8472-model1.21 | -12.5697 |
|  | XP\_065060546\_8472-model1.22 | -12.5697 |
|  | XP\_065060546\_8472-model1.23 | -12.5697 |
|  | XP\_065060546\_8472-model1.24 | -12.5697 |
|  | XP\_065060546\_8472-model1.25 | -12.5697 |
|  | XP\_065060546\_8472-model1.26 | -12.5697 |
|  | XP\_065060546\_8472-model1.27 | -12.5697 |
|  | XP\_065060546\_8472-model1.28 | -12.5697 |
|  | XP\_065060546\_8472-model1.29 | -12.5697 |
|  | XP\_065060546\_8472-model1.30 | -12.5697 |
|  | XP\_065060546\_8472-model1.31 | -12.5697 |
|  | XP\_065060546\_8472-model1.32 | -12.5697 |
|  | XP\_065060546\_8472-model1.33 | -12.5697 |
|  | XP\_065060546\_8472-model1.34 | -12.5697 |
|  | XP\_065060546\_8472-model1.35 | -12.5697 |
|  | XP\_065060546\_8472-model1.36 | -12.5697 |
|  | XP\_065060546\_8472-model1.37 | -12.5697 |
|  | XP\_065060546\_8472-model1.38 | -12.5697 |
|  | XP\_065060546\_8472-model1.39 | -12.5697 |
|  | XP\_065060546\_8472-model1.40 | -12.5697 |
|  | XP\_065060546\_8472-model1.41 | -12.5697 |
|  | XP\_065060546\_8472-model1.42 | -12.5697 |
|  | XP\_065060546\_8472-model1.43 | -12.5697 |
|  | XP\_065060546\_8472-model1.44 | -12.5697 |
|  | XP\_065060546\_8472-model1.45 | -12.5697 |
|  | XP\_065060546\_8472-model1.46 | -12.5697 |
|  | XP\_065060546\_8472-model1.47 | -12.5697 |
|  | XP\_065060546\_8472-model1.48 | -12.5697 |
|  | XP\_065060546\_8472-model1.49 | -12.5697 |
|  | XP\_065060546\_8472-model1.50 | -12.5697 |
|  | XP\_065060546\_8472-model1.51 | -12.5697 |
|  | XP\_065060546\_8472-model1.52 | -12.5697 |
|  | XP\_065060546\_8472-model1.53 | -12.5697 |
|  | XP\_065060546\_8472-model1.54 | -12.5697 |
|  | XP\_065060546\_8472-model1.55 | -12.5697 |
|  | XP\_065060546\_8472-model1.56 | -12.5697 |
|  | XP\_065060546\_8472-model1.57 | -12.5697 |
|  | XP\_065060546\_8472-model1.58 | -12.5697 |
|  | XP\_065060546\_8472-model1.59 | -12.5697 |
|  | XP\_065060546\_8472-model1.60 | -12.5697 |
|  | XP\_065060546\_8472-model1.61 | -12.5697 |
|  | XP\_065060546\_8472-model1.62 | -12.5697 |
|  | XP\_065060546\_8472-model1.63 | -12.5697 |
|  | XP\_065060546\_8472-model1.64 | -12.5697 |
|  | XP\_065060546\_8472-model1.65 | -12.5697 |
|  | XP\_065060546\_8472-model1.66 | -12.5697 |
|  | XP\_065060546\_8472-model1.67 | -12.5697 |
|  | XP\_065060546\_8472-model1.68 | -12.5697 |
|  | XP\_065060546\_8472-model1.69 | -12.5697 |
|  | XP\_065060546\_8472-model1.70 | -12.5697 |
|  | XP\_065060546\_8472-model1.71 | -12.5697 |
|  | XP\_065060546\_8472-model1.72 | -12.5697 |
|  | XP\_065060546\_8472-model1.73 | -12.5697 |
|  | XP\_065060546\_8472-model1.74 | -12.5697 |
|  | XP\_065060546\_8472-model1.75 | -12.5697 |
|  | XP\_065060546\_8472-model1.76 | -12.5697 |
|  | XP\_065060546\_8472-model1.77 | -12.5697 |
|  | XP\_065060546\_8472-model1.78 | -12.5697 |
|  | XP\_065060546\_8472-model1.79 | -12.5697 |
|  | XP\_065060546\_8472-model1.80 | -12.5697 |
|  | XP\_065060546\_8472-model1.81 | -12.5697 |
|  | XP\_065060546\_8472-model1.82 | -12.5697 |
|  | XP\_065060546\_8472-model1.83 | -12.5697 |
|  | XP\_065060546\_8472-model1.84 | -12.5697 |
|  | XP\_065060546\_8472-model1.85 | -12.5697 |
|  | XP\_065060546\_8472-model1.86 | -12.5697 |
|  | XP\_065060546\_8472-model1.87 | -12.5697 |
|  | XP\_065060546\_8472-model1.88 | -12.5697 |
|  | XP\_065060546\_8472-model1.89 | -12.5697 |
|  | XP\_065060546\_8472-model1.90 | -12.5697 |
|  | XP\_065060546\_8472-model1.91 | -12.5697 |
|  | XP\_065060546\_8472-model1.92 | -12.5697 |
|  | XP\_065060546\_8472-model1.93 | -12.5697 |
|  | XP\_065060546\_8472-model1.94 | -12.5697 |
|  | XP\_065060546\_8472-model1.95 | -12.5697 |
|  | XP\_065060546\_8472-model1.96 | -12.5697 |
|  | XP\_065060546\_8472-model1.97 | -12.5697 |
|  | XP\_065060546\_8472-model1.98 | -11.9854 |
| # cluster 2 - Size: 1 |  |  |
|  | XP\_065060546\_8472-model2 | -12.4299 |
|  |  |  |
